# Deep Hypothermic Circulatory Arrest with Lung Perfusion/Ventilation in a Patient with Acute Type A Aortic Dissection

**DOI:** 10.1155/2012/631494

**Published:** 2012-03-07

**Authors:** Yiliam F. Rodriguez-Blanco, Lester Garcia, Tania Brice, Marco Ricci, Tomas A. Salerno

**Affiliations:** ^1^Department of Anesthesiology, University of Miami Miller School of Medicine and Jackson Memorial Hospital, Miami, FL 33136, USA; ^2^Division of Cardiothoracic Surgery, University of Miami Miller School of Medicine and Jackson Memorial Hospital, Miami, FL 33136, USA

## Abstract

A 50-year-old black male presented with acute type A aortic dissection. Surgical repair was performed under deep hypothermic circulatory arrest (DHCA) with lung perfusion/ventilation throughout the procedure. Details of the lung perfusion technique and its potential benefits and drawbacks are discussed.

## 1. Introduction

Acute type A aortic dissection, involving the ascending aorta, has an incidence of 5 to 30 cases per million people per year in the United States [1]. Untreated, death usually occurs within 48 hours in 68% of patients (1%-2% deaths per hour) most commonly due to rupture of the proximal aorta into the pericardial cavity causing cardiac tamponade [2, 3]. In the event of a type A dissection, immediate surgery is indicated. The procedure usually involves replacement of the ascending aorta and reconstruction of the aortic wall by suturing the true and false lumens together, placement of a Dacron graft, and resuspension of the native aortic valve. Surgical mortality ranges from 10% to 25%, but as noted, without surgery, death is likely [4–7]. The main cause of death in those patients having a surgical repair is usually related to respiratory problems, such as ARDS with failure, pulmonary embolism, and multiple organ failure. Even in those patients who survive surgical repair, morbidity can be high with once again respiratory failure predominating [8, 9].

DHCA has become a standard of care for patients undergoing type A repairs as it facilitates the surgical procedure. However, DHCA in itself is associated with a longer duration of cardiopulmonary bypass (CPB), coagulopathy, and postoperative bleeding [10]. A major cause of morbidity and mortality associate with DHCA, as with the surgical repair of the aorta itself, is respiratory failure, which in turn leads to prolonged mechanical ventilation [8].

During CPB, with or without DHCA, lung collapse is considered a standard part of the technique. It is believed that by allowing the lungs to collapse, the overall incidence of pulmonary complications is increased. It has been proposed that there may be an advantage to ventilating the lungs of patients undergoing CPB and in particular those under DHCA.

Herein, we report the use of DHCA with lung perfusion/ventilation during the repair of an acute type A dissection. This technique's potential benefits and drawbacks are also discussed.

## 2. Case Report

A 50-year-old black male, with a history of drug addiction, was admitted with shortness of breath and peripheral edema. He denied prior medical problems and/or a history of acute chest or back pain. He had severe jugular venous distention, a 4/6 continuous diastolic murmur throughout the precordium, +3 pitting edema in the legs up to the knees, bibasilar crackles, and pulsatile hepatomegaly. Chest X-ray showed an enlarged heart with no evidence of pulmonary edema. Transthoracic echocardiography demonstrated type A aortic dissection, severe aortic and tricuspid insufficiency, and no pericardial effusion. CT angiography confirmed the diagnosis of a type A aortic dissection, beginning 2 cm from the left coronary ostium and extending to both iliac arteries. The false lumen was noted to extend into the left common carotid artery, and the left subclavian artery was occluded at its origin. The patient gave consent for the publication of this case report. The requirement for written informed consent was waived by the local IRB. 

### 2.1. Operative Technique

At the time of surgery, the central venous pressure (CVP) was noted to be 47 mmHg, and the PaO_2_ was 122 mmHg on an FiO_2_ of 100%. Transesophageal echocardiogram (TEE) confirmed the angiographic finding of a type A dissection, severe aortic and tricuspid insufficiency, and mild mitral regurgitation. Further findings demonstrated that the right atrium was notably enlarged and, the right ventricular (RV) function was decreased. The left ventricular ejection fraction was noted at 40%.

Arterial inflow was via the right subclavian artery (no. 8 mm Hemashield graft sewn to the side of the subclavian artery) and bicaval venous cannulation. CPB was instituted, and systemic cooling was initiated. The right atrium was opened, and the coronary sinus was cannulated. From the arterial cannula, a no. 8 mm polystan cannula was used to perfuse the lungs via the main pulmonary artery at flows of 300 mL/min (this is a current internal standard based on normal pulmonary artery flow rates of approximately 300–400 mL/min). The lungs were ventilated with a tidal volume of 400 mL (calculated at 4–6 mL/kg), at a rate of 8–10 breaths/min, and an FiO_2_ of 21–40% was utilized throughout the procedure. Ventilation was adjusted, if needed, based on arterial blood gases results and presence of ETCO_2_. A vent was inserted into the left atrium via the fossa ovale. The aorta was cross-clamped when ventricular fibrillation occurred at approximately 28 degrees Celsius. Cold continuous blood cardioplegia (or cold blood, once arrest occurred) was administered via the coronary sinus (pressure 50 mmHg, flows 250–300 mL/min) and simultaneously into the left coronary ostium once the aorta was opened. A tear was identified in the midportion of the ascending aorta. The false and true lumens were sewn together using a two-felt suture technique with resuspension of the aortic valve. DHCA was induced at 18 degrees Celsius. Cerebral perfusion (pressure 50 mmHg, flow 800 mL) was achieved by clamping the innominate artery. The arch was repaired using a similar technique as for the proximal aorta. A no. 33 Hemashield graft was interposed. After deairing, systemic flow was resumed, and the patient was slowly rewarmed. During this period, the tricuspid valve was repaired using a DeVega plasty. The remainder of the procedure was uneventful. The patient required minimal ionotropic support to separate from CPB. Postprocedure TEE revealed mild AI and mild TR. Blood gas measurements, immediately after weaning from CPB, showed improvement in PaO_2_/FiO_2_ ratio (Table 1), with a PaO_2_ of 579 mmHg. The patient was extubated 12 hrs postoperatively. He made an uneventful recovery and was discharged home on day 8. 

## 3. Discussion 

CPB-induced lung injury remains as an important cause of morbidity and mortality after cardiac surgery. The pathophysiology of post-CPB lung dysfunction is multifactorial, and our understanding remains incomplete despite many years of research into this phenomenon. During CPB, pulmonary artery blood flow is discontinued, and nonpulsatile flow is established, resulting in a low mean systemic perfusion pressure. Decreased bronchial artery flow is one of the etiological factors in ischemia-reperfusion injury of the lungs [11, 12]. Furthermore, lung collapse, from discontinuation of mechanical ventilation, aortic manipulation, and hypothermia, further contributes to pulmonary dysfunction, manifested by atelectasis, intrapulmonary shunting, poor gas exchange, pulmonary edema, and the need for prolonged artificial ventilation. Pulmonary artery perfusion, with or without ventilation, has been reported as a potential intervention aimed at preventing lung ischemia during CPB (12,13,14,15,16,17), and end-tidal CO_2_ has been used as an indication of pulmonary blood flow during CPB (18). 

**Figure 1:**
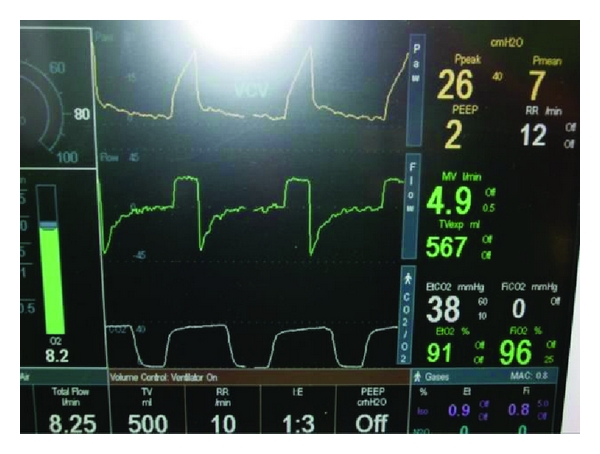
ETCO_2_ before CPB.

**Figure 2:**
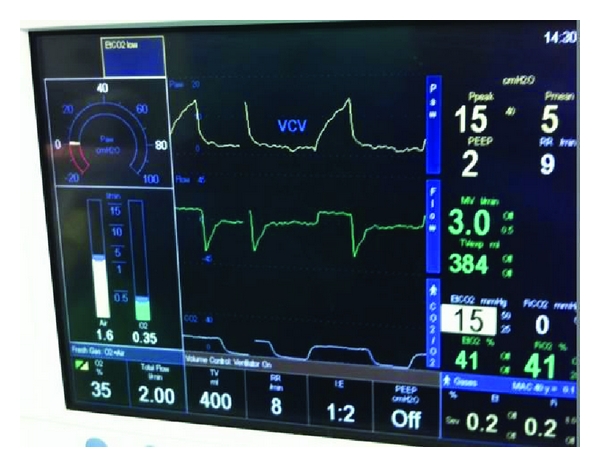
ETCO_2_ during CPB.

**Figure 3:**
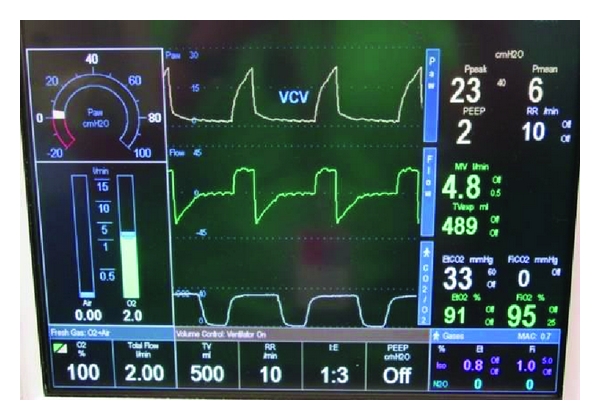
ETCO_2_ after CPB.

Lung perfusion/ventilation has been routinely used in valve surgery at our institution (13, 19) for the past 5 years; however, it has not been used during DHCA. To the best of our knowledge, this is the first case report in which lung perfusion/ventilation was utilized during DHCA in humans. Lung perfusion/ventilation during DHCA has been investigated in animal models (11, 13), demonstrating beneficial effects in terms of gas function and morphology. In our case, during the period of DHCA with ventilation and perfusion, the lungs continued to produce end-tidal CO_2_, indicating pulmonary perfusion and alveolar gas exchange (Figures 1, 2, and 3). Furthermore, as shown in Table 1, blood gases immediately after CPB showed dramatic improvement in PaO_2_ compared to baseline measurements. Although this improvement may have been related to surgical correction of the aortic and tricuspid insufficiency, better lung protection is also a possible explanation. Our clinical experience with lung perfusion/ventilation during valve surgery has shown no adverse effects, and no complications associated directly with this technique. However, whether this technique provides clinical benefits remains controversial. Much remains to be determined, such as what is the optimal pulmonary artery flow rates and whether excessive perfusion of the lungs results in pulmonary edema/hemorrhage. Matching perfusion to ventilation is another critical goal to be achieved. Ventilation/perfusion mismatch during CPB with lung perfusion/ventilation may potentially lead to dead space ventilation, shunting, barotrauma, and alveolar edema. Additionally, most of the cardiac surgeons have limited or no experience in utilizing lung perfusion/ventilation during CPB and may have issue with blood in the operative field. We hope that this paper will lead others to pursue investigation into lung perfusion/ventilation either experimentally or clinically. We continue to use this technique in all cardiac procedures in which CPB is used. 

## Figures and Tables

**Table 1:** Arterial blood gases analysis.

ABG parameters	Postanesthesia induction	Weaning from CPB	2 hours after CPB	3 hours after CPB	5 hours after CPB
PH	7.41	7.41	7.52	7.36	7.34
PCO_2_	44	41	34	43	44
PO_2_	122	579	419	580	474
HCO_3_	27.9	26	24.5	24.3	24
BE	2.7	1.2	1.9	−1.1	−1.9
SAT	99	100	100	100	100
FiO_2_	1.0	1.0	1.0	1.0	1.0
PaO_2_/FiO_2_	122	579	419	580	474
